# Perceived digital convenience and life satisfaction among older adults in China: a moderated mediation model of social adaptation and internet use

**DOI:** 10.3389/fpsyg.2026.1976506

**Published:** 2026-10-08

**Authors:** Xiagang Song, Zichuan Wang, Qiong Dang, Hua Wang

**Affiliations:** 1 School of Law, Shihezi University, Xinjiang, China; 2 School of Social and Public Administration, East China University of Science and Technology, Shanghai, China

**Keywords:** internet use, life satisfaction, older adults, perceived digital convenience, social adaptation

## Abstract

**Objective:**

This study examined the association between perceived digital convenience and life satisfaction among older adults in China, focusing on the mediating role of social adaptation and the moderating role of internet use.

**Methods:**

Using data from the 2023 China Longitudinal Aging Social Survey (CLASS), this cross-sectional study included 10,276 older adults aged 60 years and above. Mediation and moderated mediation models were estimated using the PROCESS macro (Models 4 and 7). The significance of the indirect effects and the index of moderated mediation was tested using bias-corrected bootstrap methods with 5,000 resamples.

**Results:**

Perceived digital convenience was positively associated with life satisfaction. A significant indirect association between perceived digitallk digital convenience and life satisfaction through social adaptation was observed. Moreover, internet use moderated the association between perceived digital convenience and social adaptation, such that perceived digital convenience was positively associated with social adaptation among internet users but not among non-users. Accordingly, the indirect association between perceived digital convenience and life satisfaction through social adaptation was significant among internet users but not among non-users.

**Conclusion:**

Higher levels of perceived digital convenience were associated with greater life satisfaction among older adults. Social adaptation accounted for part of this association. The indirect association was statistically significant among internet users but not among non-users. These findings underscore the importance of considering social adaptation and internet-use status together when examining the association between perceived digital convenience and life satisfaction among older adults.

## 1 Introduction

Population aging is unfolding alongside the rapid digitalization of everyday life. Digital technologies increasingly shape how older adults communicate, obtain information, access healthcare and public services, manage daily affairs, and participate in social activities (Kebede et al., 2022; Li et al., 2025). These changes may create opportunities for maintaining independence and social connectedness, but may also pose challenges when digital environments do not align with older adults' abilities and needs (Bertolazzi et al., 2024; Kebede et al., 2022). As everyday activities and essential services become increasingly mediated by digital systems, digitalization has become an important part of the social environment in which older adults pursue healthy aging (Li et al., 2025). Understanding its implications for life satisfaction, a central dimension of subjective wellbeing in later life (Diener et al., 1999), is therefore increasingly important.

As digital-divide scholarship has expanded from disparities in access to differences in technology use and outcomes (Lythreatis et al., 2022), studies of older adults have increasingly examined the implications of digital access, internet use, digital literacy, and digital skills for wellbeing (Ji et al., 2026; Kebede et al., 2022). However, technology use and the subjective experience of technology are not equivalent. Older adults' experiences of digital technology may vary substantially, ranging from enhanced autonomy and participation to frustration, dependence, and withdrawal (Akgül et al., 2026). To capture this experiential dimension, emerging research has introduced perceived digital convenience (PDC) to describe older adults' perceptions of how digital technologies facilitate or hinder different domains of everyday life (Xie et al., 2026). Using this cross-domain perspective, the present study conceptualizes PDC as older adults' subjective appraisal of the extent to which digitalization facilitates or hinders their everyday functioning. This perspective is consistent with research that distinguishes the use of digital methods in everyday activities from their perceived utility (Benge et al., 2024). Unlike perceived usefulness, which typically concerns the utility of a specific technology for achieving a particular goal (Davis, 1989), PDC captures a broader appraisal of how digitalization affects everyday functioning. Examining PDC therefore shifts attention from whether older adults are digitally connected to how they experience living in an increasingly digitalized society (Cattoni et al., 2026).

The association between PDC and life satisfaction may depend on how older adults adapt to and engage with an increasingly digitalized social environment. Social adaptation may provide a mechanism linking PDC to life satisfaction, while internet use may condition the association between PDC and social adaptation by providing opportunities for older adults to engage directly with digital resources in everyday life (Fan, 2026; Nimrod, 2020). Examining subjective appraisal, social adaptation, and behavioral engagement together may therefore provide a more comprehensive understanding of how digitalization relates to wellbeing in later life.

### 1.1 Perceived digital convenience and life satisfaction

Life satisfaction reflects individuals' overall cognitive evaluation of their life circumstances and represents an important dimension of subjective wellbeing in later life (Diener, 1984; Diener et al., 1985). Previous studies have linked digital engagement and internet use to greater life satisfaction and subjective wellbeing among older adults (Heo et al., 2015; Luo et al., 2025), suggesting that digital technologies may contribute to wellbeing when they facilitate meaningful aspects of everyday life. However, this literature has primarily operationalized digitalization in terms of access or use, offering limited insight into how older adults experience the increasingly digitalized environment in which everyday life takes place.

Evidence from related lines of research nevertheless suggests that the subjective and integrative dimensions of digitalization may be relevant to life satisfaction. Perceived accessibility to digital services has been positively associated with life satisfaction among older adults (Kim, 2024). A sense of gain from internet convenience has also been found to link smart older adult care with life satisfaction (Mao et al., 2024). Perceived functional and emotional affordances of smartphone use have similarly been associated with greater life satisfaction among older adults (Li et al., 2022). Satisfaction with changes resulting from digital transformation has also been identified as a mechanism linking digital competence to life satisfaction (Baek et al., 2025), while digital social integration has been positively associated with life satisfaction among Chinese older adults (Liu and Wu, 2026). Although these constructs differ from PDC, they indicate that the wellbeing implications of digitalization may depend not only on whether older adults use digital technologies but also on how they evaluate and integrate digitalization into their lives.

From a person-environment fit perspective, wellbeing depends partly on the degree of compatibility between individuals' needs and capacities and the demands and resources of their environment (Iwarsson et al., 2007; Lawton and Nahemow, 1973). As digital technologies become increasingly embedded in everyday life, perceived digital convenience may reflect the extent to which older adults perceive the digitalized environment as compatible with their everyday needs. When digitalization facilitates access to information and services, communication, and the management of daily affairs, older adults may experience fewer environmental barriers and greater autonomy in everyday functioning. Conversely, when digitalization is experienced as burdensome or obstructive, it may create a mismatch between older adults' needs and environmental demands (Benge et al., 2024; Cattoni et al., 2026). Greater perceived digital convenience may therefore be associated with more favorable evaluations of life circumstances and, consequently, higher life satisfaction.

### 1.2 The mediating role of social adaptation

Social adaptation refers to individuals' capacity to adjust to changes in their social environment, maintain social connections and participation, and respond effectively to the demands of everyday social life (Chen, 2008). Social adaptation is particularly relevant in later life, when individuals encounter changes in health, independence, social roles, interpersonal relationships, and everyday environments (Åberg et al., 2005; Charles and Carstensen, 2010). It is also increasingly relevant to digitalization because digital technologies shape how older adults obtain information and services, maintain relationships, and participate in everyday social life (Golant, 2026; Shuaizhi and Aziz, 2023). Older adults must therefore adapt not only to particular technologies but also to a social environment increasingly organized through digital systems.

Perceived digital convenience may be positively associated with this adaptive process. From a P–E fit perspective, PDC reflects older adults' appraisal of whether the resources and demands of the digitalized environment are compatible with their everyday needs and capacities, whereas social adaptation reflects their psychosocial adjustment within that environment. The two constructs therefore represent related but distinct components: PDC captures an evaluative appraisal of environmental compatibility, while social adaptation captures the capacity to adjust and function effectively in response to environmental change. When older adults experience digital technologies as making everyday activities easier, they may be better able to access services, maintain social connections, and participate in social life. Experiencing fewer environmental barriers may also be associated with a greater capacity to preserve autonomy, maintain established routines and social roles, and respond effectively to changing social conditions. This proposition is consistent with evidence that digital media use is positively associated with older adults' social adaptation (Shuaizhi and Aziz, 2023), as well as research showing that digital access can support online social engagement and network activities (He et al., 2020). PDC may therefore indicate a more favorable perceived fit between the demands of an increasingly digitalized environment and older adults' capacity to navigate everyday life, which may be reflected in better social adaptation. Conversely, when digitalization is experienced as obstructive, access to social resources and participation may become more difficult. Such experiences may indicate a mismatch between environmental demands and personal capacities, potentially increasing dependence on others and placing additional demands on psychosocial adjustment. In this sense, social adaptation reflects older adults' active adjustment to a changing digitalized environment rather than their passive exposure to technological change.

Social adaptation, in turn, may be positively associated with life satisfaction because better-adapted older adults may be more able to maintain social engagement, draw on valued resources, preserve meaningful roles, and manage changes in everyday life. Previous research has demonstrated that higher levels of social adaptation are associated with greater life satisfaction and subjective wellbeing among older adults (Liu and Yang, 2025; Zeng and He, 2024; Zhang and Xu, 2026). Social adaptation may therefore provide an important psychosocial pathway through which perceived digital convenience is associated with life satisfaction.

### 1.3 The moderating role of internet use

Internet use and PDC capture distinct but complementary dimensions of older adults' experiences of digitalization. PDC reflects a subjective appraisal of whether digitalization facilitates or hinders everyday life, whereas internet use reflects behavioral engagement with digital resources and services. A favorable appraisal of the digital environment does not necessarily produce adaptive benefits unless the opportunities associated with that environment are translated into actual behavior. Drawing on the general logic of affordance actualization, technology-related opportunities generate outcomes through the actions by which users realize them in practice (Strong et al., 2014).

Internet use may therefore condition whether perceived digital convenience can be translated into adaptive benefits. Evidence suggests that the implications of internet use for older adults depend on how digital technologies are incorporated into communication, information seeking, and other meaningful activities (Benvenuti et al., 2020). Technology-based interventions may also support older adults' social connectedness by providing additional channels for interaction, although their effects vary across technologies and contexts (Balki et al., 2022). Among internet users, perceived digital convenience may therefore be more readily translated into effective everyday functioning and social adaptation through actual engagement with digital resources. Among non-users, limited behavioral engagement may constrain this translation.

Internet use may consequently strengthen the positive association between PDC and social adaptation. Because social adaptation is positively associated with life satisfaction, the indirect association between PDC and life satisfaction through social adaptation may also be stronger among internet users than among non-users.

Taken together, these perspectives form a complementary, sequential, and conditional framework rather than three independent explanations assigned to separate statistical paths. P-E fit provides the overarching person-in-environment perspective: PDC reflects older adults' appraisal of the compatibility between the demands and resources of an increasingly digitalized environment and their own needs and capacities. Social adaptation specifies the psychosocial process through which this perceived fit may be translated into broader wellbeing. When older adults perceive digitalization as compatible with their needs, they may be better able to maintain everyday functioning, access social resources, preserve valued roles, and adjust to changing social conditions, thereby supporting greater life satisfaction. Affordance actualization further identifies a behavioral boundary condition on this process. Perceiving the digital environment as convenient does not necessarily improve adaptation unless the opportunities it offers can be realized through actual engagement. Internet use therefore represents a behavioral channel through which perceived digital affordances may be enacted in everyday life. Within this framework, older adults are not treated as passive recipients of technological change: their agency is reflected in how they appraise the digital environment, adjust to its demands, and engage with its opportunities.

### 1.4 The present study

Building on the preceding literature, the present study examines the association between perceived digital convenience and life satisfaction among older adults, with particular attention to the mediating role of social adaptation and the moderating role of internet use. The proposed conceptual model is presented in Figure 1. Accordingly, we propose the following hypotheses:

- H1: Perceived digital convenience is positively associated with life satisfaction among older adults.
- H2: Social adaptation mediates the association between perceived digital convenience and life satisfaction.
- H3: Internet use moderates the association between perceived digital convenience and social adaptation.
- H4: Internet use moderates the indirect association between perceived digital convenience and life satisfaction through social adaptation.

**Figure 1:**
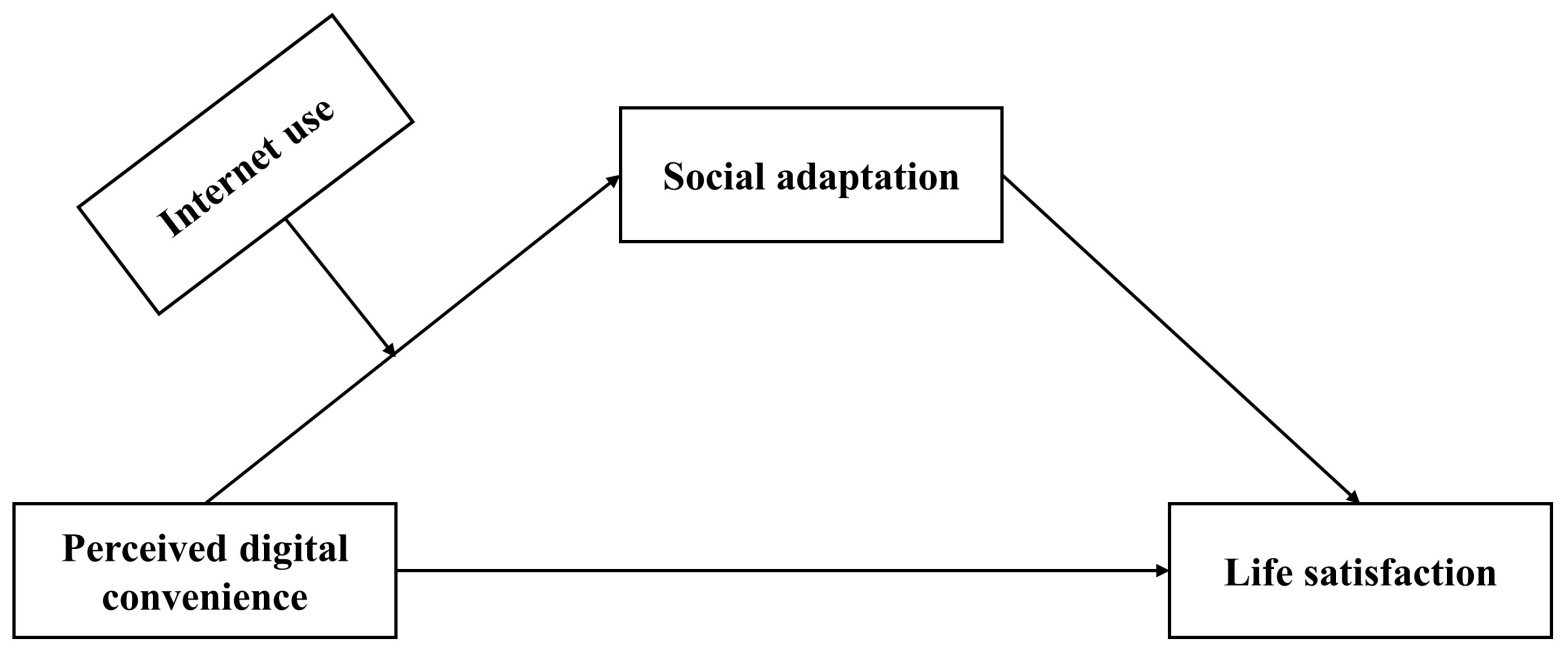
The theoretical model of the study.

## 2 Materials and methods

### 2.1 Data source and participants

Data were obtained from the 2023 China Longitudinal Aging Social Survey (CLASS), a nationwide longitudinal social survey of older adults in China. CLASS is administered by the Population Development Studies Center at Renmin University of China and collects comprehensive information on older adults' health, family circumstances, social backgrounds, and economic conditions.

The 2023 survey was conducted by trained interviewers using a standardized Chinese questionnaire. The questionnaire provided item-specific instructions for interviewers, and subjective items, including life satisfaction, were required to be answered by the sampled respondents rather than proxy respondents. Fieldwork quality-control procedures included supervisory review and field or telephone verification.

The 2023 CLASS included 11,670 respondents. We excluded 22 respondents younger than 60 years, 58 with missing data on life satisfaction, 83 with missing data on perceived digital convenience, 238 with missing data on social adaptation, and 993 with missing data on one or more covariates. After these exclusions, the final analytic sample comprised 10,276 participants.

### 2.2 Measures

#### 2.2.1 Life satisfaction

Life satisfaction was measured using a single item asking participants to rate their overall satisfaction with their current life. Responses were originally rated on a five-point scale from 1 (very satisfied) to 5 (very dissatisfied) and were subsequently reverse-coded, yielding scores ranging from 1 to 5, with higher scores indicating greater life satisfaction.

#### 2.2.2 Perceived digital convenience

Consistent with recent research (Xie et al., 2026), perceived digital convenience was assessed using 10 items from the CLASS questionnaire. Respondents were asked, “What impact have internet technologies commonly used in society today had on the following aspects of your life?” The 10 items covered: “social interaction;” “shopping and consumption;” “obtaining news and information”; “leisure and entertainment”; “travel and tourism”; “access to health services”; “investment and financial management”; “learning and training”; “payment of public service fees, such as water, electricity, gas, and social security”; and “participation in community affairs.” For each item, respondents selected one of four response options: “convenient,” “inconvenient,” “no impact,” or “difficult to judge.” Responses were recoded as 1 for “convenient,” −1 for “inconvenient,” and 0 for “no impact,” whereas “difficult to judge” responses were treated as missing. PDC was operationalized as a formative cross-domain composite because the items represent distinct domains that jointly characterize PDC. In the absence of respondent-provided domain-importance ratings or a validated external weighting scheme, the available valid items were equally weighted and averaged. The resulting score ranged from −1 to 1, with higher scores indicating greater perceived digital convenience.

#### 2.2.3 Social adaptation

Social adaptation was measured using the eight-item Social Adaptation Scale (Chen, 2008), which has been widely applied in empirical studies in the Chinese context (Fan, 2026; Liu and Yang, 2025; Song and Tian, 2026). The scale comprised the following eight items: “If given the opportunity, I would be willing to participate in some of the work of the village or neighborhood committee”; “I often wish to contribute to society again”; “I enjoy learning”; “I believe that I am still useful to society”; “Society is changing so rapidly that I find it difficult to adapt to these changes”; “I find it difficult to accept the increasing diversity of viewpoints nowadays”; “I find it difficult to accept the growing number of new social policies nowadays”; and “Current social changes are becoming increasingly unfavorable to older adults.” Respondents rated how closely each statement reflected their current situation on a five-point Likert scale ranging from 1 (not at all applicable) to 5 (completely applicable). The last four items were negatively worded and reverse-coded. The social adaptation score was calculated as the mean of the eight items, with higher scores indicating greater social adaptation. The scale demonstrated acceptable internal consistency in the present study (Cronbach's alpha = 0.76).

#### 2.2.4 Internet use

Internet use was assessed by asking participants how often they accessed the internet via mobile phones or other electronic devices. Response options were daily, at least once a week, at least once a month, several times a year, and never. Participants reporting any internet use were coded as 1 and classified as internet users, whereas those reporting no internet use were coded as 0 and classified as non-users.

#### 2.2.5 Covariates

Covariates were selected to account for individual differences in demographic, socioeconomic, household, sociocultural, and health characteristics. Demographic characteristics included age in years, interviewer-recorded sex (male = 1, female = 0), ethnicity (ethnic minority = 1, Han = 0), and current marital status (married with a spouse = 1; widowed, divorced, or never married = 0). Socioeconomic indicators included years of education, annual personal income, and hukou status. Years of education were derived from respondents' highest level of educational attainment. Annual personal income referred to respondents' total personal income during the preceding 12 months and was log-transformed to reduce right skewness. Hukou status was coded as agricultural hukou = 1 and non-agricultural hukou = 0. Living alone was determined from the reported number of people regularly living and eating together in the household (yes = 1, no = 0). Sociocultural characteristics included political status (other political status = 1, general public = 0) and religious belief (yes = 1, no = 0). Chronic disease status indicated whether respondents reported having at least one of the chronic diseases listed in the questionnaire (yes = 1, no = 0). Descriptive statistics for all study variables are presented in Table 1.

**Table 1:** Descriptive statistics (*N* = 10,276).

Variables	M/*n*	SD/%	Min	Max
Life satisfaction	3.81	0.751	1	5
Perceived digital convenience	0.271	0.443	−1	1
Social adaptation	3.033	0.519	1	5
Internet use				
Non-user	5,100	49.63%		
Internet user	5,176	50.37%		
Age (years)	71.432	5.987	60	101
Sex				
Female	4,924	47.92%		
Male	5,352	52.08%		
Ethnicity				
Han	9,533	92.77%		
Ethnic minority	743	7.23%		
Marital status				
Widowed, divorced, or never married	1,784	17.36%		
Married with a spouse	8,492	82.64%		
Years of education	6.49	3.874	0	16
Log personal income	9.527	1.181	4.605	12.899
Hukou status				
Non-agricultural	4,772	46.44%		
Agricultural hukou	5,504	53.56%		
Living alone				
No	9,385	91.33%		
Yes	891	8.67%		
Political status				
General public	9,965	96.97%		
Other political status	311	3.03%		
Religious belief				
No	9,875	96.10%		
Yes	401	3.90%		
Chronic disease status				
No	1,785	17.37%		
Yes	8,491	82.63%		

M, Mean; *n* = frequency; SD, Standard Deviation.

### 2.3 Statistical analysis

Statistical analyses were conducted using SPSS version 26.0 and the PROCESS macro for SPSS (Hayes, 2018). Descriptive statistics and Pearson correlation analyses were then performed for the main study variables. Mediation analyses were conducted using PROCESS Model 4 to examine whether social adaptation mediated the association between perceived digital convenience and life satisfaction. Moderated mediation analyses were performed using PROCESS Model 7 to test whether internet use moderated the first-stage path from perceived digital convenience to social adaptation. Given the cross-sectional nature of the data, these analyses were interpreted as assessing statistical indirect and conditional associations consistent with the proposed theoretical model, rather than establishing temporal ordering or causal relationships. All models were adjusted for the covariates described above. Variance inflation factors ranged from 1.02 to 1.75, indicating no serious multicollinearity concerns. Ordinary least squares regression was used, and indirect effects were estimated using bias-corrected bootstrapping with 5,000 resamples. Effects were considered statistically significant if the 95% bootstrap confidence interval did not include zero. Conditional effects and conditional indirect effects were estimated separately for internet users and non-users.

In the primary moderated mediation analysis, internet use was entered as a binary moderator to distinguish respondents who reported any direct internet use from those who reported no internet use. The binary specification was used because the focal theoretical distinction was whether respondents had any opportunity to realize perceived digital affordances through their own direct internet use, rather than the frequency or intensity of that engagement. To examine whether the findings were sensitive to this dichotomization, two alternative specifications were estimated using the original frequency information. First, internet-use frequency was treated as an ordinal moderator. Second, respondents were classified into three categories: frequent users, infrequent users, and non-users. Frequent users reported using the internet daily or at least once a week; infrequent users reported using it at least once a month or several times a year; and non-users reported never using the internet.

Several robustness and sensitivity analyses were also conducted. First, the models were re-estimated using the survey weights provided by CLASS. Second, perceived digital convenience was reconstructed using a principal component score and an alternative binary measure in which “convenient” was coded as 1 and “inconvenient” or “no impact” as 0. A domain-specific analysis was also conducted using the healthcare-services item. Finally, because “difficult to judge” responses were treated as missing and the primary PDC score was calculated from the available items, a complete-item sensitivity analysis was conducted by restricting the sample to respondents with valid responses to all 10 PDC items and complete data on the remaining model variables.

## 3 Results

### 3.1 Descriptive statistics and correlations

Table 1 presents the descriptive statistics of all study variables. The analytic sample comprised 10,276 older adults. The mean life satisfaction score was 3.81 (SD = 0.75), with scores ranging from 1 to 5. The mean perceived digital convenience score was 0.27 (SD = 0.44), with scores ranging from −1 to 1, and the mean social adaptation score was 3.03 (SD = 0.52), with scores ranging from 1 to 5. Approximately half of the participants (50.37%) were internet users. The mean age of the sample was 71.43 years (SD = 5.99), with a range of 60–101 years, and 52.08% were male. Most participants were of Han ethnicity (92.77%) and married with a spouse (82.64%). The mean number of years of education was 6.49 years (SD = 3.87), and the mean log-transformed personal income was 9.53 (SD = 1.18). In addition, 53.56% held agricultural hukou, 8.67% lived alone, 3.03% had other political status, and 3.90% reported a religious belief. Overall, 82.63% of the participants reported having at least one chronic disease.

Supplementary Table 1 presents perceived digital convenience, social adaptation, and life satisfaction across population subgroups. Internet users reported higher mean levels of all three variables than non-users. Higher mean levels of perceived digital convenience, social adaptation, and life satisfaction were also generally observed among respondents who were married with a spouse, held non-agricultural hukou, or were not living alone. In addition, respondents without chronic diseases reported higher life satisfaction than those with chronic diseases.

Table 2 presents the correlations among the key study variables. Perceived digital convenience was positively correlated with life satisfaction (*r* = 0.118, *p* < 0.001) and social adaptation (*r* = 0.173, *p* < 0.001). Social adaptation was also positively correlated with life satisfaction (*r* = 0.166, *p* < 0.001). In addition, internet use was positively correlated with life satisfaction (*r* = 0.152, *p* < 0.001), perceived digital convenience (*r* = 0.397, *p* < 0.001), and social adaptation (*r* = 0.208, *p* < 0.001). These correlations provide preliminary support for the hypothesized associations among perceived digital convenience, social adaptation, internet use, and life satisfaction.

**Table 2:** Correlation analysis of key variables.

Variables	Life satisfaction	Perceived digital convenience	Social adaptation	Internet use
Life satisfaction	1			
Perceived digital convenience	0.118^***^	1		
Social adaptation	0.166^***^	0.173^***^	1	
Internet use	0.152^***^	0.397^***^	0.208^***^	1

^***^p < 0.001.

### 3.2 Association between perceived digital convenience and life satisfaction

Table 3 presents the results of the linear regression models examining the association between perceived digital convenience and life satisfaction. Model 1 included the covariates only, whereas Model 2 additionally included perceived digital convenience. After adjustment for all covariates, perceived digital convenience was positively associated with life satisfaction (*B* = 0.164, *p* < 0.001), indicating that older adults who perceived greater convenience from digital technologies tended to report higher levels of life satisfaction. These results supported Hypothesis 1.

**Table 3:** Linear regression models examining the association between perceived digital convenience and life satisfaction.

Variables	Model 1 Life satisfaction	Model 2 Life satisfaction
Perceived digital convenience		0.164^***^ (0.017)
Age	−0.000 (0.001)	0.001 (0.001)
Sex	−0.005 (0.015)	−0.006 (0.015)
Ethnicity	0.055 (0.029)	0.030 (0.028)
Marital status	0.106^***^ (0.025)	0.105^***^ (0.025)
Years of education	0.007^***^ (0.002)	0.007^**^ (0.002)
Log personal income	0.051^***^ (0.007)	0.044^***^ (0.007)
Hukou status	−0.082^***^ (0.016)	−0.071^***^ (0.016)
Living alone	−0.056 (0.033)	−0.059 (0.033)
Political status	0.037 (0.043)	0.040 (0.043)
Religious belief	−0.060 (0.038)	−0.061 (0.038)
Chronic disease status	−0.241^***^ (0.019)	−0.250^***^ (0.019)
Constant	3.466^***^ (0.131)	3.381^***^ (0.131)
*N*	10276	10276
*R* ^2^	0.039	0.048

Values are unstandardized regression coefficients (B), with standard errors in parentheses. Reference categories were female, Han ethnicity, widowed, divorced, or never married, non-agricultural hukou, not living alone, general public, no religious belief, and no chronic disease. ^**^p < 0.01, ^***^p < 0.001.

### 3.3 Mediation analysis

Mediation analysis was conducted using PROCESS Model 4 to examine whether social adaptation mediated the association between perceived digital convenience and life satisfaction. As shown in Table 4, perceived digital convenience was positively associated with social adaptation (*B* = 0.163, *p* < 0.001). In turn, social adaptation was positively associated with life satisfaction after controlling for perceived digital convenience (*B* = 0.188, *p* < 0.001). Perceived digital convenience also remained positively associated with life satisfaction (*B* = 0.134, *p* < 0.001). As shown in Table 5, the total effect of perceived digital convenience on life satisfaction was significant (effect = 0.164, p < 0.001). Bootstrap analysis further showed that the indirect effect of perceived digital convenience on life satisfaction through social adaptation was significant [indirect effect = 0.031, 95% CI (0.024, 0.038)]. These results supported Hypothesis 2.

**Table 4:** Regression models examining the mediating role of social adaptation.

Variables	Model 3 Social adaptation	Model 4 Life satisfaction
Perceived digital convenience	0.163^***^ (0.012)	0.134^***^ (0.017)
Social adaptation		0.188^***^ (0.014)
Age	−0.003^***^ (0.001)	0.002 (0.001)
Sex	−0.014 (0.010)	−0.003 (0.015)
Ethnicity	0.006 (0.020)	0.029 (0.028)
Marital status	0.000 (0.017)	0.106^***^ (0.025)
Years of education	0.014^***^ (0.002)	0.004 (0.002)
Log personal income	0.031^***^ (0.005)	0.038^***^ (0.007)
Hukou status	−0.025^*^ (0.011)	−0.066^***^ (0.016)
Living alone	−0.031 (0.022)	−0.054 (0.032)
Political status	0.082^**^ (0.030)	0.024 (0.043)
Religious belief	−0.066^*^ (0.026)	−0.049 (0.037)
Chronic disease status	−0.033^*^ (0.013)	−0.244^***^ (0.019)
Constant	2.885^***^ (0.090)	2.838^***^ (0.136)
*N*	10276	10276
*R* ^2^	0.061	0.064

Values are unstandardized regression coefficients (B), with standard errors in parentheses. Reference categories were female, Han ethnicity, widowed, divorced, or never married, non-agricultural hukou, not living alone, general public, no religious belief, and no chronic disease. ^*^p < 0.05, ^**^p < 0.01, ^***^p < 0.001.

**Table 5:** Total, direct, and indirect effects of perceived digital convenience on life satisfaction.

Effect	Estimate	SE	95% CI	
			Lower	Upper
Total effect	0.164^***^	0.017	0.131	0.197
Direct effect	0.134^***^	0.017	0.100	0.167
Indirect effect	0.031	0.003	0.024	0.038

Values are unstandardized effects. For the indirect effect, SE and 95% CI were estimated using 5,000 bias-corrected bootstrap resamples. Covariates were included in the analysis. ^***^p < 0.001.

### 3.4 Moderated mediation analysis

A moderated mediation analysis was conducted using PROCESS Model 7 to examine whether internet use moderated the first-stage path from perceived digital convenience to social adaptation. As shown in Table 6, the interaction between perceived digital convenience and internet use was positively associated with social adaptation (*B* = 0.194, *p* < 0.001), indicating that internet use significantly moderated the association between perceived digital convenience and social adaptation. To further interpret this interaction, conditional effects were examined separately for non-users and internet users. Among non-users, perceived digital convenience was not significantly associated with social adaptation (*B* = 0.007, *p* = 0.714). In contrast, among internet users, perceived digital convenience was positively associated with social adaptation (*B* = 0.202, *p* < 0.001). These interaction effects are illustrated in Figure 2. These results supported Hypothesis 3.

**Table 6:** Moderating effect of internet use on the association between perceived digital convenience and social adaptation.

Variables	Model 5 Social adaptation
Perceived digital convenience	0.007 (0.020)
Internet use	0.067^***^ (0.014)
Perceived digital convenience × Internet use	0.194^***^ (0.025)
Age	−0.001 (0.001)
Sex	−0.012 (0.010)
Ethnicity	0.017 (0.019)
Marital status	−0.009 (0.017)
Years of education	0.012^***^ (0.002)
Log personal income	0.022^***^ (0.005)
Hukou status	−0.010 (0.011)
Living alone	−0.027 (0.022)
Political status	0.081^**^ (0.029)
Religious belief	−0.066^*^ (0.026)
Chronic disease status	−0.042^**^ (0.013)
Constant	2.781^***^ (0.090)
*N*	10276
*R* ^2^	0.074

Values are unstandardized regression coefficients (B), with standard errors in parentheses. Reference categories were female, Han ethnicity, widowed, divorced, or never married, non-agricultural hukou, not living alone, general public, no religious belief, and no chronic disease. ^*^p < 0.05, ^**^p < 0.01, ^***^p < 0.001.

**Figure 2:**
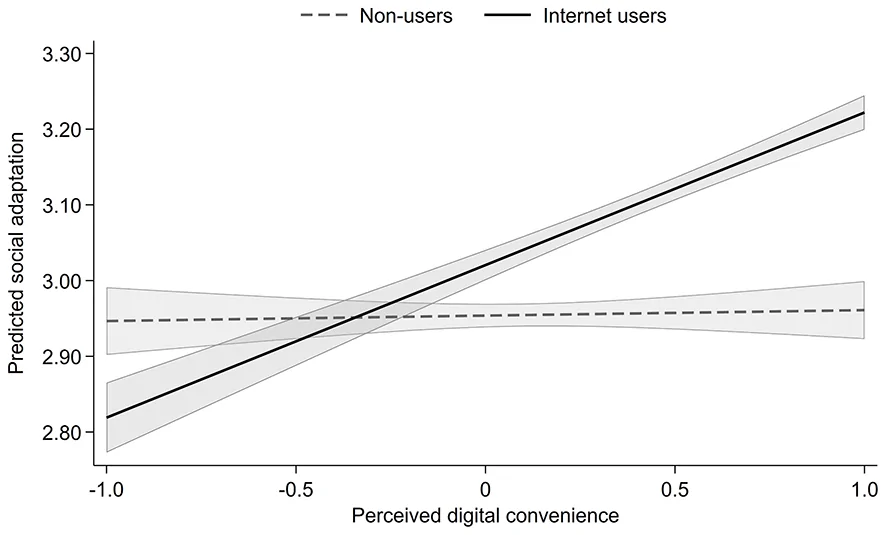
Moderating effect of internet use on the association between perceived digital convenience and social adaptation.

As shown in Table 7, the conditional indirect effect of perceived digital convenience on life satisfaction through social adaptation was not significant among non-users [indirect effect = 0.001, 95% CI (−0.006, 0.008)], whereas it was significant among internet users [indirect effect = 0.038, 95% CI (0.030, 0.047)]. The index of moderated mediation was significant [index = 0.037, 95% CI (0.027, 0.049)], indicating that the indirect effect of perceived digital convenience on life satisfaction through social adaptation differed according to internet use. These results supported Hypothesis 4.

**Table 7:** Conditional indirect effects of perceived digital convenience on life satisfaction through social adaptation by internet use.

Effect	Estimate	Boot SE	95% bootstrap CI
Conditional indirect effects			
Non-users	0.001	0.004	[−0.006, 0.008]
Internet users	0.038	0.005	[0.030, 0.047]
**Index of moderated mediation**	0.037	0.006	[0.027, 0.049]

Bootstrap standard errors and 95% confidence intervals were estimated using 5,000 bias-corrected bootstrap resamples. Covariates were included in the analysis. Boot SE, bootstrap standard error; CI, confidence interval.

### 3.5 Additional robustness and sensitivity analyses

Supplementary Table 3 presents the results of the survey-weighted analysis. perceived digital convenience was positively associated with life satisfaction (*B* = 0.167, *p* < 0.001), and the interaction between perceived digital convenience and internet use was positively associated with social adaptation (*B* = 0.184, *p* < 0.001).

Supplementary Tables 4A–C present the results obtained using alternative operationalizations of perceived digital convenience. As shown in Supplementary Table 4A, when perceived digital convenience was constructed using a principal component score, perceived digital convenience was positively associated with life satisfaction (*B* = 0.039, *p* < 0.001), and the interaction between perceived digital convenience and internet use was positively associated with social adaptation (*B* = 0.055, *p* < 0.001). As shown in Supplementary Table 4B, when “convenient” was coded as 1 and “inconvenient” or “no impact” as 0, the alternatively recoded perceived digital convenience measure was positively associated with life satisfaction (*B* = 0.186, *p* < 0.001), and its interaction with internet use was positively associated with social adaptation (*B* = 0.105, *p* < 0.001). As shown in Supplementary Table 4C, perceived convenience in healthcare services was positively associated with life satisfaction (*B* = 0.068, *p* < 0.001), and its interaction with internet use was positively associated with social adaptation (*B* = 0.109, *p* < 0.001).

Supplementary Tables 5A, B present the results obtained using alternative specifications of internet use. As shown in Supplementary Table 5A, when internet-use frequency was treated as a continuous moderator, the interaction between perceived digital convenience and internet-use frequency was positively associated with social adaptation (*B* = 0.057, *p* < 0.001). As shown in Supplementary Table 5B, the interaction between perceived digital convenience and frequent internet use was positive and statistically significant (*B* = 0.199, *p* < 0.001), whereas the interaction between perceived digital convenience and infrequent internet use was not statistically significant (*B* = −0.091, *p* > 0.05).

Supplementary Table 2 shows that “difficult to judge” responses, which were treated as missing, ranged from 8.98 to 14.59% across the 10 perceived digital convenience domains. To assess the sensitivity of the findings to variation in the number of valid perceived digital convenience items, the models were re-estimated among respondents with valid responses to all 10 items and complete data on the remaining variables (*N* = 5,624). Supplementary Table 4D shows that perceived digital convenience remained positively associated with life satisfaction (*B* = 0.188, *p* < 0.001), and that the interaction between perceived digital convenience and internet use remained positively associated with social adaptation (*B* = 0.264, *p* < 0.001). Supplementary Table 6 presents the conditional associations. The association between perceived digital convenience and social adaptation was not statistically significant among non-users (*B* = 0.035, *p* = 0.165), but was positive and statistically significant among internet users (*B* = 0.298, *p* < 0.001). Although the coefficient among non-users was slightly larger than that observed in the primary analysis (*B* = 0.007), it remained small and statistically nonsignificant. This modest numerical difference likely reflects differences in sample composition and in the number of valid items contributing to the PDC score and does not alter the substantive interpretation of the conditional pattern. Supplementary Table 7 shows that the index of moderated mediation also remained statistically significant [index = 0.023, 95% CI (0.011, 0.035)]. Overall, these results indicate that the principal findings were not driven by variation in the number of valid perceived digital convenience items used to calculate respondents' scores.

## 4 Discussion

Using data from the 2023 China Longitudinal Aging Social Survey (CLASS), the present study examined the association between older adults' perceived digital convenience and life satisfaction, as well as the mediating role of social adaptation and the moderating role of internet use. The results revealed four main findings. First, perceived digital convenience was positively associated with life satisfaction. Second, social adaptation partially mediated this association. Third, internet use moderated the association between perceived digital convenience and social adaptation, with a significant positive association observed among internet users but not among non-users. Fourth, the indirect association between perceived digital convenience and life satisfaction through social adaptation was significant among internet users but not among non-users. Taken together, these findings point to an integrated process in which older adults' appraisal of the digitalized environment is associated with life satisfaction both directly and indirectly through social adaptation, while internet use conditions the indirect pathway.

### 4.1 Perceived digital convenience and life satisfaction

Perceived digital convenience was positively associated with life satisfaction among older adults. This finding is consistent with previous research associating both a greater sense of gain from internet convenience and perceived technology affordances with higher life satisfaction among older adults (Li et al., 2022; Mao et al., 2024). Satisfaction with changes resulting from digital transformation has also been identified as an important mechanism linking digital competence to life satisfaction (Baek et al., 2025). Moreover, digital social integration has been positively associated with life satisfaction among older adults (Liu and Wu, 2026). Taken together, these findings highlight the potential relevance of older adults' subjective experiences of digitalization to their life satisfaction.

The present study extends this emerging literature by conceptualizing perceived digital convenience as a cross-domain appraisal of whether digitalization facilitates or hinders everyday functioning. Older adults who perceived digital technologies as facilitating communication, information acquisition, healthcare access, financial management, travel, consumption, and other daily activities reported greater life satisfaction. Previous studies have reported inconsistent associations between internet or digital technology use and life satisfaction among older adults (Aggarwal et al., 2020; Grintal et al., 2026; Luo et al., 2025). These inconsistencies underscore the limitations of relying solely on indicators of digital access or use to understand older adults' wellbeing. Whether older adults use the internet does not fully capture how they experience the broader digitalization of everyday life. Perceived digital convenience may therefore capture an aspect of older adults' digital experiences that is not adequately represented by indicators of digital access or use alone. By shifting attention from behavioral engagement to the perceived implications of digitalization for everyday functioning, this study provides a complementary perspective for understanding its relevance to life satisfaction.

Viewed within the integrated framework, perceived digital convenience may reflect older adults' perceived fit with an increasingly digitalized environment. From a person–environment fit perspective, greater perceived digital convenience may indicate that the demands and resources of this environment are experienced as more compatible with older adults' everyday needs and capacities (Iwarsson et al., 2007; Lawton and Nahemow, 1973). When digitalization is experienced as convenient, it may reduce perceived environmental barriers, expand available options, and strengthen autonomy and control over everyday affairs, thereby contributing to more favorable evaluations of life circumstances. At the same time, the mediating and moderating findings indicate that the association between perceived digital convenience and life satisfaction cannot be understood solely as a direct association. Social adaptation provides an additional psychosocial pathway, while internet use conditions the extent to which perceived digital convenience is associated with such adaptation. These interrelated findings are discussed in the following sections.

### 4.2 The mediating role of social adaptation

Social adaptation partially mediated the association between perceived digital convenience and life satisfaction. Greater perceived digital convenience was associated with better social adaptation, which was in turn associated with higher life satisfaction. Within the integrated framework, this finding positions social adaptation as a potential psychosocial process through which older adults' perceived fit with the digitalized environment may be connected to their broader evaluations of life.

Perceived digital convenience may be positively associated with social adaptation because it indicates that older adults experience the digitalized environment as facilitating rather than hindering their everyday functioning. When digital technologies are experienced as facilitating access to information and services, communication, and the management of daily affairs, older adults may encounter fewer difficulties in maintaining everyday routines, accessing social resources, and participating in social life. Existing evidence indicates that digital technologies can facilitate everyday activities, social connectedness, and participation among older adults (Kebede et al., 2022). These experiences may support their ability to adjust to changing social conditions and function effectively within contemporary society. Conversely, when digitalization is experienced as obstructive, difficulties in accessing essential services and participating in everyday activities may place additional demands on social adaptation. Qualitative research has similarly documented that digital technologies can enrich older adults' daily lives and social relationships while also generating frustration, dependence, and a need for assistance when they are difficult to use (Emmesjö et al., 2025).

Better social adaptation may, in turn, be associated with life satisfaction because it enables older adults to maintain social engagement, preserve meaningful roles, draw on valued resources, and manage changes in everyday life. The positive association between social adaptation and life satisfaction is consistent with longitudinal evidence linking social adaptation to subjective wellbeing among older adults (Fan, 2026). Greater social interaction has also been associated with higher life satisfaction among older adults (Park and Kang, 2023). Adaptation to losses in activity and independence has also been shown to help older adults maintain life satisfaction (Åberg et al., 2005). The present findings extend this evidence by indicating that social adaptation may also connect older adults' experiences of the digitalized environment with their broader evaluations of life. Taken together, these findings suggest that social adaptation may connect older adults' experiences of the digitalized environment with life satisfaction by helping them maintain everyday functioning, social engagement, and valued roles amid technological change.

### 4.3 The moderating role of internet use

Internet use moderated the association between perceived digital convenience and social adaptation. Perceived digital convenience was positively associated with social adaptation among internet users but not among non-users. Consistent with this pattern, the indirect association between perceived digital convenience and life satisfaction through social adaptation was also significant among internet users but not among non-users. These findings identify the behavioral condition under which perceived compatibility with the digitalized environment may be translated into adaptive benefits. This conditional pattern is consistent with evidence that internet use can alter the relationship between older adults' personal resources and psychological outcomes (Roquet et al., 2024). Related research has also shown that the psychological benefits associated with internet use depend on whether older adults possess the skills needed to engage effectively with digital resources (Yang and Jang, 2024).

Within the integrated framework, perceived digital convenience and internet use represent distinct but complementary components of older adults' experiences of digitalization. Perceived digital convenience reflects a subjective appraisal of whether digitalization facilitates or hinders everyday life, whereas internet use reflects behavioral engagement with digital resources and services. A favorable appraisal of the digital environment does not necessarily produce adaptive benefits unless the opportunities associated with that environment are translated into actual behavior. Drawing on the general logic of affordance actualization, technology-related opportunities generate outcomes through the actions by which users realize them in practice (Strong et al., 2014). Internet use may therefore provide a behavioral channel through which perceived person-environment compatibility becomes relevant to everyday functioning and social adaptation.

Evidence suggests that the implications of internet use for older adults depend on how digital technologies are incorporated into communication, information seeking, and other meaningful activities (Benvenuti et al., 2020). Technology-based interventions may also support older adults' social connectedness by providing additional channels for interaction, although their effects vary across technologies and contexts (Balki et al., 2022). Internet users can directly use digital channels to obtain information and services, communicate with others, maintain social relationships, and perform everyday activities. Such engagement may allow the opportunities reflected in perceived digital convenience to be translated into greater access to resources, participation in social life, and more effective everyday functioning. Longitudinal evidence further indicates that the wellbeing implications of internet use vary according to the purposes for which it is used, reinforcing the importance of how digital resources are incorporated into everyday activities (Szabo et al., 2019). The present findings extend this evidence by suggesting that behavioral engagement may also condition whether a favorable appraisal of digitalization is associated with social adaptation.

The nonsignificant association between perceived digital convenience and social adaptation among non-users does not necessarily mean that their perceptions of digitalization are irrelevant. Non-users may recognize the general benefits of digitalization without being able to incorporate those benefits directly into their own everyday activities. Some may also rely on family members or others to access digital services on their behalf. Although such indirect access may help them complete particular tasks, it may not provide the same opportunities for independent engagement and adaptation. Previous research has shown that assistance from partners can make online services accessible to older adults while, in some circumstances, reinforcing dependence and reducing motivation to develop independent digital skills (Marler and Hargittai, 2024). Limited opportunities for direct engagement may therefore provide one possible explanation for the weaker association between perceived digital convenience and social adaptation among non-users.

However, the difference between internet users and non-users should be interpreted cautiously because the experiential basis of perceived digital convenience ratings may differ between the two groups. Internet users may evaluate perceived digital convenience primarily through direct engagement with digital technologies, whereas non-users may rely on indirect exposure, proxy-assisted access, anticipated consequences, or experiences of exclusion from digitalized services. Among non-users, a response of “no impact” may also indicate either a genuinely neutral appraisal or that a particular digitalized domain was not personally applicable. These possibilities cannot be distinguished using the available items. Consequently, the weaker association between perceived digital convenience and social adaptation among non-users may partly reflect differential measurement error or differences in the meaning of the perceived digital convenience measure, rather than only a substantive difference in the strength of the association.

Taken together, the findings indicate that the association between digitalization and later-life wellbeing varies according to internet-use status. Older adults' appraisal of whether the digitalized environment is compatible with their needs and capacities was directly associated with life satisfaction. It was also indirectly associated with life satisfaction through social adaptation among internet users but not among non-users. However, the observed difference between the two groups may reflect both variation in behavioral engagement and possible differences in how perceived digital convenience is interpreted and measured. Subject to this measurement-related qualification, P-E fit, social adaptation, and affordance actualization illuminate interconnected components of the same conceptual framework rather than providing separate explanations for individual statistical paths.

### 4.4 Practical implications

The findings suggest that efforts to promote older adults' wellbeing in a digitalized society should combine age-friendly digital environments with support for internet access and use. Digital inclusion should involve not only creating digital environments that older adults perceive as convenient but also providing the access, skills, and support needed to use digital resources in everyday life (Hunsaker et al., 2019). Governments and communities should therefore go beyond expanding digital access by offering task-oriented training and sustained technical assistance, particularly to older adults with limited digital skills or experience. Public service providers and technology developers should simplify digital interfaces and procedures, involve older adults in their design and evaluation, and preserve accessible offline alternatives for those who cannot or prefer not to use the internet. Community organizations could incorporate digital guidance into social activities and peer-support programs, helping older adults use digital technologies for communication, service access, and everyday tasks. Whenever possible, family members should provide explanations and guided practice rather than routinely completing digital tasks on older adults' behalf, while recognizing that proxy assistance remains necessary for some individuals. Together, these measures may reduce barriers to internet use, support everyday functioning and social adaptation, and safeguard older adults' autonomy, choice, and dignity.

### 4.5 Limitations and future research

The study has several limitations that may inform future research. First, the cross-sectional design does not establish the temporal ordering among perceived digital convenience, social adaptation, internet use, and life satisfaction. Reverse associations are possible because older adults with better social adaptation or greater life satisfaction may evaluate digitalization more favorably and may also be more likely to use the internet. Although the analyses adjusted for a range of demographic, socioeconomic, household, and health-related characteristics, residual confounding by unmeasured individual and contextual factors, such as functional health, social support, and local digital infrastructure, cannot be ruled out. The indirect and conditional associations identified in the present study should therefore be interpreted as statistical relationships rather than as evidence of causal processes. Future longitudinal studies using multiwave data and temporally separated measurements are needed to clarify the direction and temporal ordering of these associations.

Second, life satisfaction was assessed using a single item in CLASS. Although single-item measures provide a concise assessment, they may be more susceptible to measurement error and may not capture the construct as comprehensively as validated multi-item measures. In addition, the focal variables were primarily based on self-reported information collected within the same survey, and shared method variance may therefore have influenced the observed associations. The available data did not include a prespecified marker variable, and the combination of multi-item and single-item measures did not permit comparable latent method-factor modeling across all focal constructs. Because CLASS does not provide a suitable validated multi-item measure of life satisfaction or a marker variable designed to assess method effects, these limitations could not be addressed within the current dataset. Future studies should use validated multi-item measures of life satisfaction, draw on longitudinal or multiple-source data, and incorporate appropriate methodological controls for shared method variance.

Third, perceived digital convenience was operationalized as a survey-derived formative cross-domain composite based on 10 CLASS items rather than as an independently validated scale. Because the items represent distinct domains that jointly constitute perceived digital convenience, conventional reflective CFA was not considered appropriate. The equal-weight composite does not account for differences in the personal importance of the assessed domains and allows perceived convenience in one domain to offset perceived inconvenience in another. It should therefore be interpreted as an average cross-domain appraisal rather than as an individualized, importance-weighted evaluation. Although the findings remained consistent across alternative scoring methods and the complete-item sample, these analyses demonstrate operational robustness rather than establishing construct validity. In addition, CLASS did not include validated measures of related constructs against which the convergent and discriminant validity of perceived digital convenience could be assessed. The experiential basis and interpretation of the PDC items may differ between internet users and non-users, and cross-group measurement equivalence could not be established using the available data. Consequently, the weaker association observed among non-users may partly reflect differential measurement error rather than solely a substantive difference in the association between PDC and social adaptation. The conditional findings should therefore be interpreted cautiously. Future research should further examine the conceptual boundaries, measurement properties, domain-specific importance, and cross-group comparability of perceived digital convenience, distinguish direct experience, proxy-assisted access, indirect exposure, and non-applicability, and develop validated weighting schemes where appropriate.

Fourth, internet use was represented by a binary indicator that classified all respondents reporting any use as internet users. This approach does not distinguish between occasional and frequent use or capture the purposes, duration, intensity, skills, quality, and outcomes of online engagement. It also does not identify older adults who access digital services indirectly through family members or other proxy users. Consequently, the moderating effect should be interpreted as a difference between respondents reporting any direct internet use and those reporting none rather than as evidence concerning specific forms of digital engagement. Future studies should employ multidimensional measures that distinguish access, frequency, purpose, digital skills, independent use, and proxy use to determine which aspects of internet engagement condition the association between perceived digital convenience and social adaptation.

Fifth, although the principal findings remained substantively consistent after applying the sampling weights provided by CLASS, the weighted sensitivity analyses did not incorporate all elements of the complex survey design, including stratification and clustering. The results should therefore be interpreted as sampling-weighted robustness estimates rather than as fully design-based population estimates and should not be assumed to be nationally representative of all older adults in China. Moreover, the study used complete-case analysis, which may have introduced selection bias if missingness was systematically related to observed or unobserved respondent characteristics.

Finally, because the study was conducted among older adults in China, the observed associations may be shaped by the country's particular technological and social context. The rapid expansion of mobile payment and digitally mediated public and commercial services, uneven digital infrastructure across urban and rural areas, differences in the availability of accessible offline alternatives, and older adults' reliance on family members for proxy-assisted access may influence both how digital convenience is perceived and how internet use is associated with social adaptation and life satisfaction. These contextual features, together with China's welfare arrangements and patterns of family support, may differ from those in other societies. The findings, particularly the conditional associations involving internet use, should therefore be generalized cautiously. Replication in countries and cultural contexts with different levels and forms of digitalization is needed to assess their broader applicability.

## 5 Conclusion

The present study found that perceived digital convenience was positively associated with life satisfaction among older adults and that social adaptation statistically accounted for part of this association. Internet use further conditioned this indirect association. Perceived digital convenience was associated with better social adaptation and indirectly with higher life satisfaction among internet users but not among non-users. These findings highlight the importance of distinguishing perceived digital convenience, as a subjective appraisal of the digitalized environment, from internet use, as an indicator of direct behavioral engagement with digital resources. Efforts to support life satisfaction in an increasingly digitalized society may therefore benefit from both the development of convenient and age-responsive digital environments and the provision of practical support for older adults' use of digital resources in everyday life.

## Data Availability

Publicly available datasets were analyzed in this study. This data can be found at: http://class.ruc.edu.cn.
