# Supplementary material for: Perceived digital convenience and life satisfaction among older adults in China: a moderated mediation model of social adaptation and internet use
Source: Front Psychol. 2026 Oct 8;17:1976506. doi: 10.3389/fpsyg.2026.1976506 (PMC13687368; doi:10.3389/fpsyg.2026.1976506)
Supplement: Supplementary file 1 [file Data_Sheet_1.pdf]

Table S1. Perceived digital convenience, social adaptation, and life satisfaction across population subgroups

Variables	n (%)	Perceived digital convenience, M (SD)	Social adaptation, M (SD)	Life satisfaction, M (SD)
Internet use				
Non-user	5,100 (49.63%)	0.094 (0.357)	2.924 (0.481)	3.695 (0.769)
Internet user	5,176 (50.37%)	0.445 (0.449)	3.140 (0.532)	3.923 (0.715)
Sex				
Female	4,924 (47.92%)	0.263 (0.440)	3.027 (0.509)	3.794 (0.758)
Male	5,352 (52.08%)	0.279 (0.445)	3.038 (0.528)	3.824 (0.744)
Ethnicity				
Han	9,533 (92.77%)	0.263 (0.441)	3.034 (0.523)	3.809 (0.752)
Ethnic minority	743 (7.23%)	0.368 (0.449)	3.016 (0.468)	3.813 (0.737)
Marital status				
Widowed, divorced, or never married	1,784 (17.36%)	0.208 (0.418)	2.964 (0.503)	3.662 (0.781)
Married with a spouse	8,492 (82.64%)	0.284 (0.446)	3.047 (0.521)	3.841 (0.741)
Hukou status				
Non-agricultural	4,772 (46.44%)	0.330 (0.456)	3.092 (0.516)	3.882 (0.733)
Agricultural hukou	5,504 (53.56%)	0.220 (0.425)	2.982 (0.516)	3.747 (0.761)
Living alone				
No	9,385 (91.33%)	0.276 (0.445)	3.041 (0.520)	3.825 (0.745)
Yes	891 (8.67%)	0.219 (0.412)	2.952 (0.501)	3.646 (0.796)
Political status				
General public	9,965 (96.97%)	0.270 (0.443)	3.028 (0.518)	3.806 (0.748)
Other political status	311 (3.03%)	0.306 (0.432)	3.194 (0.517)	3.920 (0.829)
Religious belief				
No	9,875 (96.10%)	0.270 (0.441)	3.036 (0.519)	3.812 (0.745)
Yes	401 (3.90%)	0.302 (0.472)	2.967 (0.502)	3.761 (0.879)
Chronic disease status				
No	1,785 (17.37%)	0.242 (0.447)	3.062 (0.496)	4.004 (0.733)
Yes	8,491 (82.63%)	0.277 (0.441)	3.027 (0.523)	3.769 (0.748)
Note: Values are presented as n (%) or M (SD). M = mean; SD = standard deviation.				

Supplementary Table S1 presents perceived digital convenience, social adaptation, and life satisfaction across population subgroups. Compared with non-users, internet users reported higher perceived digital convenience, social adaptation, and life satisfaction. Higher levels of the three variables were also generally observed among respondents who were married, held non-agricultural or resident hukou, or not living alone. Respondents without chronic diseases reported higher life satisfaction than those with chronic diseases.

Table S2. Distribution of responses across the ten domains of perceived digital convenience

Domain	Convenient	Inconvenient	No impact	Difficult to judge
Social interaction	5,573 (54.23%)	711 (6.92%)	2,997 (29.17%)	995 (9.68%)
Shopping and consumption	4,665 (45.40%)	1,456 (14.17%)	3,027 (29.46%)	1,128 (10.98%)
Information acquisition	5,090 (49.53%)	1,361 (13.24%)	2,902 (28.24%)	923 (8.98%)
Entertainment	4,364 (42.47%)	1,160 (11.29%)	3,376 (32.85%)	1,376 (13.39%)
Transportation and travel	3,727 (36.27%)	1,220 (11.87%)	3,914 (38.09%)	1,415 (13.77%)
Healthcare services	3,567 (34.71%)	1,565 (15.23%)	3,960 (38.54%)	1,184 (11.52%)
Financial management	2,789 (27.14%)	1,452 (14.13%)	4,589 (44.66%)	1,446 (14.07%)
Learning and training	2,680 (26.08%)	1,474 (14.34%)	4,623 (44.99%)	1,499 (14.59%)
Public services and payments	4,402 (42.84%)	1,530 (14.89%)	3,176 (30.91%)	1,168 (11.37%)
Community participation	3,107 (30.24%)	1,614 (15.71%)	4,201 (40.88%)	1,354 (13.18%)

Note: Values are presented as n (%). Percentages were calculated based on the total analytic sample (N = 10,276). Responses of "difficult to judge" were treated as missing values when constructing the perceived digital convenience index.

Table S3. Robustness analysis using survey-weighted estimation

Variables	Life satisfaction	Social adaptation
Perceived digital convenience	0.167*** (0.018)	0.005 (0.020)
Internet use		0.057*** (0.016)
Perceived digital convenience × Internet use		0.184*** (0.026)
Age	-0.001 (0.002)	-0.004*** (0.001)
Sex	-0.002 (0.016)	-0.008 (0.011)
Ethnicity	0.025 (0.031)	0.019 (0.019)
Marital status	0.117*** (0.032)	-0.001 (0.020)
Years of education	0.004 (0.002)	0.012*** (0.002)
Log personal income	0.043*** (0.007)	0.017*** (0.005)
Hukou status	-0.092*** (0.018)	-0.040** (0.013)
Living alone	-0.041 (0.041)	-0.012 (0.025)
Political status	0.013 (0.054)	0.071* (0.032)
Religious belief	-0.046 (0.045)	-0.058* (0.026)
Chronic disease status	-0.230*** (0.021)	-0.037** (0.014)
Constant	3.554*** (0.145)	3.042*** (0.097)
N	10276	10276
R^2	0.048	0.075

Note: The models were estimated using the survey weights provided by CLASS. Values are unstandardized regression coefficients (*B*), with standard errors in parentheses. Reference categories were female, Han ethnicity,widowed, divorced, or never married, non-agricultural hukou, not living alone, general public, no religious belief, and no chronic disease. * $p < 0.05$, ** $p < 0.01$, *** $p < 0.001$.

Table S4A. Robustness analysis using a principal component analysis-based measure of perceived digital convenience

Variables	Life satisfaction	Social adaptation
Perceived digital convenience	0.039*** (0.004)	0.008 (0.005)
Internet use		0.144*** (0.017)
Perceived digital convenience × Internet use		0.055*** (0.007)
Age	0.000 (0.002)	0.000 (0.001)
Sex	-0.002 (0.018)	-0.004 (0.012)
Ethnicity	0.048 (0.031)	0.025 (0.022)
Marital status	0.052 (0.033)	-0.003 (0.023)
Years of education	0.007* (0.003)	0.010*** (0.002)
Log personal income	0.032*** (0.009)	0.036*** (0.006)
Hukou status	-0.121*** (0.021)	0.027 (0.015)
Living alone	-0.070 (0.042)	-0.036 (0.029)
Political status	0.012 (0.056)	0.084* (0.038)
Religious belief	-0.132** (0.050)	-0.067 (0.035)
Chronic disease status	-0.216*** (0.025)	0.007 (0.017)
Constant	3.688*** (0.157)	2.505*** (0.110)
N	5624	5624
R^2	0.068	0.128

Note: The variable "Perceived digital convenience" was reconstructed using principal component analysis (PCA) based on ten domain-specific indicators as an alternative measure for sensitivity analysis. Values are unstandardized regression coefficients (*B*), with standard errors in parentheses. Reference categories were female, Han ethnicity,widowed, divorced, or never married, non-agricultural hukou, not living alone, general public, no religious belief, and no chronic disease. * $p < 0.05$, ** $p < 0.01$, *** $p < 0.001$.

Table S4B. Robustness analysis using an alternatively recoded measure of perceived digital convenience

Variables	Life satisfaction	Social adaptation
Perceived digital convenience	0.186*** (0.019)	0.097*** (0.025)
Internet use		0.094*** (0.015)
Perceived digital convenience × Internet use		0.105*** (0.029)
Age	0.001 (0.001)	-0.001 (0.001)
Sex	-0.006 (0.015)	-0.012 (0.010)
Ethnicity	0.034 (0.028)	0.013 (0.019)
Marital status	0.105*** (0.025)	-0.008 (0.017)
Years of education	0.007*** (0.002)	0.012*** (0.002)
Log personal income	0.043*** (0.007)	0.021*** (0.005)
Hukou status	-0.076*** (0.016)	-0.011 (0.011)
Living alone	-0.063 (0.033)	-0.028 (0.022)
Political status	0.038 (0.043)	0.080** (0.029)
Religious belief	-0.063 (0.038)	-0.068** (0.026)
Chronic disease status	-0.244*** (0.019)	-0.040** (0.013)
Constant	3.383*** (0.131)	2.755*** (0.090)
N	10276	10276
R^2	0.048	0.075

Note: The variable "Perceived digital convenience" was reconstructed by coding "convenient" as 1 and "inconvenient" or "no impact" as 0 and then calculating the mean across the available items. Values are unstandardized regression coefficients (*B*), with standard errors in parentheses. Reference categories were female, Han ethnicity,widowed, divorced, or never married, non-agricultural hukou, not living alone, general public, no religious belief, and no chronic disease. $^{*}p < 0.05$, $^{**}p < 0.01$, $^{***}p < 0.001$.

Table S4C. Sensitivity analysis using perceived convenience in the healthcare-services domain

Variables	Life satisfaction	Social adaptation
Health service convenience	0.068*** (0.011)	0.001 (0.011)
Internet use		0.115*** (0.013)
Health service convenience × Internet use		0.109*** (0.015)
Age	-0.000 (0.001)	-0.001 (0.001)
Sex	-0.009 (0.015)	-0.011 (0.011)
Ethnicity	0.073* (0.029)	0.024 (0.020)
Marital status	0.103*** (0.026)	0.001 (0.018)
Years of education	0.006** (0.002)	0.011*** (0.002)
Log personal income	0.049*** (0.007)	0.027*** (0.005)
Hukou status	-0.081*** (0.017)	-0.009 (0.012)
Living alone	-0.044 (0.034)	-0.003 (0.023)
Political status	0.040 (0.045)	0.072* (0.031)
Religious belief	-0.105** (0.040)	-0.068* (0.027)
Chronic disease status	-0.243*** (0.020)	-0.024 (0.014)
Constant	3.487*** (0.136)	2.718*** (0.094)
N	9092	9092
R^2	0.046	0.075

Note: Values are unstandardized regression coefficients (*B*), with standard errors in parentheses. Reference categories were female, Han ethnicity,widowed, divorced, or never married, non-agricultural hukou, not living alone, general public, no religious belief, and no chronic disease. * $p < 0.05$, ** $p < 0.01$, *** $p < 0.001$.

Table S4D. Sensitivity analysis restricted to respondents with valid responses to all ten perceived digital convenience items

Variables	Life satisfaction	Social adaptation
Perceived digital convenience	0.188*** (0.020)	0.035 (0.025)
Internet use		0.031 (0.019)
Perceived digital convenience × Internet use		0.264*** (0.031)
Age	0.000 (0.002)	0.000 (0.001)
Sex	-0.002 (0.018)	-0.004 (0.012)
Ethnicity	0.048 (0.031)	0.025 (0.022)
Marital status	0.052 (0.033)	-0.003 (0.023)
Years of education	0.007* (0.003)	0.010*** (0.002)
Log personal income	0.032*** (0.009)	0.036*** (0.006)
Hukou status	-0.121*** (0.021)	0.027 (0.015)
Living alone	-0.070 (0.042)	-0.036 (0.029)
Political status	0.013 (0.056)	0.085* (0.039)
Religious belief	-0.132** (0.050)	-0.067 (0.035)
Chronic disease status	-0.216*** (0.025)	0.007 (0.017)
Constant	3.607*** (0.158)	2.490*** (0.110)
N	5,624	5,624
R^2	0.068	0.129

Note: The analytic sample excluded respondents who selected "difficult to judge" on any of the ten PDC items, as well as respondents with missing data on other variables. Values are unstandardized regression coefficients (*B*), with standard errors in parentheses. Reference categories were female, Han ethnicity,widowed, divorced, or never married, non-agricultural hukou, not living alone, general public, no religious belief, and no chronic disease. * $p < 0.05$, ** $p < 0.01$, *** $p < 0.001$.

Table S5A. Robustness analysis treating internet-use frequency as a continuous moderator

Variables	Social adaptation
Perceived digital convenience	-0.012 (0.019)
Internet use	0.018*** (0.004)
Perceived digital convenience × Internet use	0.057*** (0.006)
Age	-0.000 (0.001)
Sex	-0.012 (0.010)
Ethnicity	0.019 (0.019)
Marital status	-0.011 (0.017)
Years of education	0.012*** (0.002)
Log personal income	0.021*** (0.005)
Hukou status	-0.007 (0.011)
Living alone	-0.028 (0.022)
Political status	0.080** (0.029)
Religious belief	-0.067** (0.026)
Chronic disease status	-0.041** (0.013)
Constant	2.762*** (0.090)
N	10276
R^2	0.076
Note: Values are unstandardized regression coefficients (*B*), with standard errors in parentheses. Reference categories were female, Han ethnicity,widowed, divorced, or never married, non-agricultural hukou, not living alone, general public, no religious belief, and no chronic disease. * $p < 0.05$, ** $p < 0.01$, *** $p < 0.001$.	

Table S5B. Robustness analysis using a three-category measure of internet use

Variables	Social adaptation
Perceived digital convenience	0.007 (0.020)
Infrequent users	0.052 (0.046)
Frequent users	0.068*** (0.014)
Perceived digital convenience × Infrequent users	-0.091 (0.101)
Perceived digital convenience ×Frequent users	0.199*** (0.025)
Age	-0.001 (0.001)
Sex	-0.013 (0.010)
Ethnicity	0.017 (0.019)
Marital status	-0.010 (0.017)
Years of education	0.012*** (0.002)
Log personal income	0.022*** (0.005)
Hukou status	-0.009 (0.011)
Living alone	-0.028 (0.022)
Political status	0.079** (0.029)
Religious belief	-0.067** (0.026)
Chronic disease status	-0.042** (0.013)
Constant	2.774*** (0.090)
N	10276
R^2	0.075

Note: Internet use was categorized as non-use (never), infrequent use (at least once a month or several times a year), and frequent use (daily or at least once a week). Non-users served as the reference group. Values are unstandardized regression coefficients (*B*), with standard errors in parentheses. Reference categories were female, Han ethnicity,widowed, divorced, or never married, non-agricultural hukou, not living alone, general public, no religious belief, and no chronic disease. * $p < 0.05$, ** $p < 0.01$, *** $p < 0.001$.

Supplementary Table S6. Conditional effects of perceived digital convenience on social adaptation in the complete-item sensitivity analysis

Internet use	Effect	SE	*t*	*p*	95% CI
Non-users	0.035	0.025	1.389	0.165	[−0.014, 0.084]
Internet users	0.298	0.019	16.064	<0.001	[0.262, 0.335]

Note: The analysis was restricted to respondents who provided valid responses to all ten PDC items and had complete data on the other model variables (N = 5,624). Respondents who selected "difficult to judge" or had a missing response on any PDC item were excluded. Effects are unstandardized conditional effects. Covariates were included in the analysis. CI = confidence interval.

Supplementary Table S7. Conditional indirect effects of perceived digital convenience on life satisfaction through social adaptation in the complete-item sensitivity analysis

Effect	Estimate	Boot SE	95% CI
**Conditional indirect effects**			
Non-users	0.003	0.002	[−0.001, 0.008]
Internet users	0.026	0.007	[0.013, 0.039]
**Index of moderated mediation**	0.023	0.006	[0.011, 0.035]

Note: The analysis was restricted to respondents who provided valid responses to all ten PDC items and had complete data on the other model variables (N = 5,624). Respondents who selected "difficult to judge" or had a missing response on any PDC item were excluded. Bootstrap standard errors and 95% confidence intervals were estimated using 5,000 bias-corrected bootstrap resamples. Covariates were included in the analysis. Boot SE = bootstrap standard error; CI = confidence interval.
